# Traditional Japanese Formula Kigikenchuto Accelerates Healing of Pressure-Loading Skin Ulcer in Rats

**DOI:** 10.1155/2011/592791

**Published:** 2011-05-16

**Authors:** Mari Kimura, Naotoshi Shibahara, Hiroaki Hikiami, Toshiko Yoshida, Michiko Jo, Maria Kaneko, Tatsuya Nogami, Makoto Fujimoto, Hirozo Goto, Yutaka Shimada

**Affiliations:** ^1^Department of Japanese Oriental Medicine, Graduate School of Medicine and Pharmaceutical Sciences, University of Toyama, 2630 Sugitani, Toyama 930-0194, Japan; ^2^Division of Kampo Diagnostics, Institute of Natural Medicine, University of Toyama, 2630 Sugitani, Toyama 930-0194, Japan; ^3^Department of Regenerative Medicine, Graduate School of Medicine and Pharmaceutical Sciences, University of Toyama, 2630 Sugitani, Toyama 930-0194, Japan

## Abstract

We evaluated the effect of kigikenchuto (KKT), a traditional Japanese formula, in a modified rat pressure-loading skin ulcer model. Rats were divided into three groups, KKT extract orally administered (250 or 500 mg/kg/day for 35 days) and control. KKT shortened the duration until healing. Immunohistochemically, KKT increased CD-31-positive vessels in early phase and increased *α*-smooth muscle actin-(*α*-SMA-) positive fibroblastic cells in early phase and decreased them in late phase of wound healing. By Western blotting, KKT showed the potential to decrease inflammatory cytokines (MCP-1, IL-1*β*, and TNF-*α*) in early phase, decrease vascular endothelial growth factor in early phase and increase it in late phase, and modulate the expression of extracellular protein matrix (*α*-SMA, TGF-*β*1, bFGF, collagen III, and collagen I). These results suggested the possibility that KKT accelerates pressure ulcer healing through decreases of inflammatory cytokines, increase of angiogenesis, and induction of extracellular matrix remodeling.

## 1. Introduction

Japan is now on its way to becoming an aging society, a situation also seen in other advanced countries. In these aging societies, geriatric diseases are necessarily receiving more and more attention, and one of such diseases is pressure ulcer (decubitus) [1, 2]. This disease has a decidedly negative impact on the quality of life. Its cause is the continuation of pressure against subcutaneous soft tissue, and external factors such as shear stress, tissue deformation, temperature, and humidity, play important roles in its generation [3–5]. Pressure ulcers are classified into two types, shallow ulcer, characterized by damage to the dermis, and deep ulcer, with damage through subcutaneous tissue. The latter is more difficult to treat compared to the former, and effective treatment for it has been awaited. Deep ulcer is defined as ulcer extending through the skin surface layer to deeper tissue. According to the grade classification of the European Pressure Ulcer Advisory Panel, grade III is damage to subcutaneous tissue, and grade IV is damage reaching to muscle or bone [6].

 Recently, a new pressure ulcer animal model utilizing magnetic compression was developed, with focus on ischemia-reperfusion injury for the formation of skin ulcerations [7, 8], and it has been used for understanding the role of ischemia-reperfusion injury in the skin [9–11]. However, this pressure ulcer animal model with magnet is not appropriate for animal models of severe pressure ulcer, because prolonged compression by magnet influences only the superficial skin but not deep muscle. Thus, to create severe pressure ulcers, various kinds of original devices have been developed as pressure indenters [12–14], but their manufacturing is not easy and their production costs are high. Then, in their stead, a simple pressure loading method using a stainless steel cylinder for 24 hours has been applied for the purpose of therapeutic drug development for severe pressure ulcers [15]. 

 Cell growth factors and angiogenesis factors are responsible for the healing process of pressure ulcers as well as wound healing, and they are divided into three main overlapping phases—inflammation, granulation tissue formation, and matrix formation and remodeling [16, 17]. The chronic wound is associated with increased infiltration of inflammatory cells, decreased production of cytokines responsible for angiogenesis, poor granulation, keloid formation due to reduced or excess accumulation of extracellular matrix, and delayed new skin formation. 

 For the treatment of pressure ulcers, growth factor products are clinically applied externally [18–21], and for oral administration there are a few supplements such as zinc preparation, vitamin C, and arginine [22–24]. In recent years, traditional Japanese (Kampo) medicines have been used for the treatment of various kinds of diseases. Clinical reports regarding Kampo medicines for the treatment of pressure ulcers have been published, and we also reported that elderly patients were successfully treated with a Kampo formula, kigikenchuto (KKT) [25, 26]. KKT is a formula created originally by a Japanese medical doctor, *Seishu Hanaoka* (1760–1835), and it has been used for the treatment of intractable skin ulcer, like pressure ulcer, in Japan. However, the active mechanism of KKT on pressure ulcer has not been clarified. The purpose of this study is to evaluate the efficacy of KKT on pressure ulcer by using a modified pressure-loading skin ulcer model of rats [15] and also to clarify its active mechanism by immunohistochemical and Western blot analyses.

## 2. Materials and Methods

### 2.1. Preparation of Extract

Seven crude drugs composing KKT, Cinnamomi Cortex (*Cinnamomum cassia* Blume), Paeoniae Radix (*Paeonia lactiflora *Pallas), Zingiberis Rhizoma (*Zingiber officinale* Roscoe), Zizyphi Fructus (*Zizyphus jujuba* var. *inermis* Rehder), Glycyrrhizae Radix (*Glycyrrhiza glabra* Fischer), Angelicae Radix (*Angelica acutiloba* Kitagawa), and Astragali Radix (*Astragalus membranaceus* Bunge), were purchased from Tochimoto Tenkaido Co. Ltd. (Osaka, Japan), and mixed at a ratio of 4 : 6 : 1 : 4 : 2 : 4 : 4. The extract was prepared by boiling a mixture (100 g) of the above crude drugs gently in 500 ml of water for 50 min and then filtrating the decoction. The filtrate was concentrated under reducing pressure, resulting in a yield of 20.4% by weight of the original preparation.

**Figure 1:**
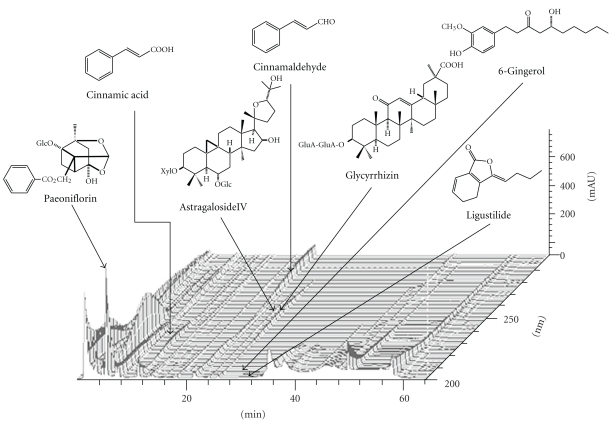
3D-HPLC profile of kigikenchuto. The peaks of guiding compounds included in each crude-drug component of kigikenchuto— paeoniflorin, cinnamic acid, cinnamaldehyde, astragaloside IV, glycyrrhizin, 6-gingerol, ligustilide, and so forth,—are presented.

 A three-dimensional high performance liquid chromatography (3D-HPLC) profile of KKT extract is shown in Figure 1. The high performance liquid chromatography (HPLC) conditions were as follows: KKT extract (0.2 g) was dissolved in 2 ml 70% acetonitrile under ultrasonication. The solution was filtered with membrane filter (0.45 *μ*m pore size; Millipore Corp., Billerica, Mass) prior to injection. The HPLC apparatus was a SD-8020 system (Tosoh Corp., Tokyo, Japan) consisting of a CCPM-II multisolvent delivery pump and a PD-8020 photodiode array detector. A TSK gel ODS-80TS column (4.6 × 150 mm i.d., 5 *μ*m, Tosoh Corp.) was used for analysis. The gradient elution was in accordance with that reported previously [27]. Briefly, the mobile phase consisted of (A) 0.017% phosphoric acid and (B) acetonitrile. The gradient elution was programmed as follows: 0–5 min, 18–20% B; 5–9 min, 20–25% B; 9–15 min, 25–29% B; 15–18 min, 29-29% B; 18–26 min, 39–43% B; 26–34 min, 43–65% B; 34–40 min, 65-65% B. The other analysis conditions were as follows: flow rate, 1.0 ml/min; injection volume, 10 *μ*l; UV scan 200–300 nm.

### 2.2. Animals and Pressure-Loading Skin Ulcer

Wistar rats weighing 410–480 g were purchased at 23 weeks of age from Japan SLC, Inc. (Shizuoka, Japan) and were kept in an automatically controlled room (temperature about 24°C and humidity about 60%) with a conventional dark/light cycle. The animals were allowed free access to standard laboratory food. All experimentation was performed in accordance with the Guide Principles for the Care and Use of Laboratory Animals approved by the Japanese Pharmacological Society and also approved by the Committee on Animal Experimentation, University of Toyama (no. S-2010 INM-4).

**Figure 2:**
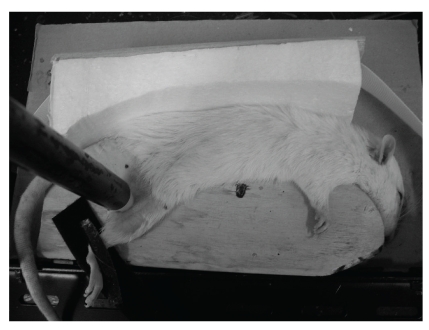
Photograph of pressure loading. The anesthetized rats were fixed in a lateral position on a wooden fixation plate. As a cushion, a piece of absorbent cotton was placed between the femoral region and the fixation plate, and a stainless steel cylinder weighing 1.02–1.03 kg and measuring 19 mm and 50 cm in diameter and length, respectively, with a silicon stopper measuring 10 mm in diameter, was placed on the skin of the right greater trochanter (1.30 to 1.31 kg/cm^2^).

 In this study we adopted a pressure ulcer model of rats [15] using our original modification. After test-keeping for one week, hair from the right greater trochanter of the rat was extensively removed using electric hair clippers and an electric shaver. For anesthesia, sodium pentobarbital at 50 mg/kg was intraperitoneally administered. Before pressure loading, rats assigned to this purpose were anesthetized, and maintenance doses of 40 mg/kg were given at 1 h 15 min and again at 3 h later. If necessary, additional anesthesia (40 mg/kg) was given during pressure loading. The anesthetized rats were fixed in a lateral position on a wooden fixation plate. As a cushion, a piece of absorbent cotton was placed between the femoral region and the fixation plate, and a stainless steel cylinder weighing 1.02–1.03 kg and measuring 19 mm and 50 cm in diameter and length, respectively, with a silicon stopper measuring 10 mm in diameter, was placed on the skin of the right greater trochanter (1.30 to 1.31 kg/cm^2^) (Figure 2). The duration of pressure loading was 6 h, and pressure loading was performed daily for 4 days.

### 2.3. Grouping and Drug Treatment

Rats that had been subjected to pressure loading for 4 days were divided into three groups, the low-dose KKT extract (250 mg/kg/day) administered group (LD-KKT), the high-dose KKT extract (500 mg/kg/day) administered group (HD-KKT), and the water-only administered group (control group). KKT extract was dissolved in 2 ml of water and administered orally through a stomach tube daily for 35 days from the last pressure-loading day. The day when pressure loading was completed was defined as day 0, the next day 1, and so forth.

### 2.4. Macroscopic Observation of Pressure Ulcer

Eighteen rats, six rats per group, were used. A picture of pressure ulcer with a caliper square and a color scale was taken daily from day 0 to day 35 by electronic camera, Optio750Z (HOYA Corp., Tokyo, Japan). The distance between the camera and skin ulcer was fixed at 23 cm. After the color image was compensated based on a standard gray of color scale by the use of Color Dial Photo 3 (Mitsubishi Electric Micro-Computer Application Software Co., Ltd., Amagasaki, Japan), the size of the ulcer image data was unified. The pressure ulcer area was calculated by image analysis software, VH Analyzer (Keyence Corp., Osaka, Japan), and the pressure ulcer area ratio, percentage of ulcer area of each day to that of day 0, was calculated. Healing of ulcer was defined as when the image analysis software could not detect ulcer.

### 2.5. Histopathological and Immunohistochemical Analysis

Eighty-four rats, seven at each evaluation day in each group, were used. At each evaluation day, rats were euthanized under anesthesia and skin ulcer areas (2.5 × 2.5 cm) were excised. Samples of skin were cut out from the head side to the tail side. Half amounts of skin ulcer tissues from days 3, 7, 14, and 35 were fixed in 4% paraformaldehyde, processed routinely, and embedded in paraffin. Five-*μ*m sections were conventionally stained with hematoxylin-eosin (HE). The paraffin-embedded specimens were deparaffinized in xylene and dehydrated with ethanol. KN9 was used for antigen retrieval (95°C, 40 min, Pathology Institute, Toyama, Japan), and endogenous peroxidase was blocked with 3% hydrogen peroxide (Kanto Chemical Industry Co., Ltd., Tokyo, Japan) for 10 min at room temperature, and then washed with distilled water and TBS. Nonspecific binding was blocked by treatment with a special blocking reagent (Dako Cytomation Co., Glostrup, Denmark) for 15 min. Anti VEGF (1 : 40; Immuno-Biological Laboratories Co., Ltd., Fujioka, Japan) [28], *α*-SMA (1 : 100; DAKO Cytomation) [29], or CD-31 (1 : 100; Santa Cruz Biotechnology Inc., CA) [30] antibody was applied, and the sections were then incubated in a moist box for 30 min. After washing the specimens with TBS, they were then stained using a commercial kit (ChemMate EnVision Detection System Peroxidase/HRP; DAKO Cytomation). After counterstaining by hematoxylin, sections were rinsed, dehydrated, and covered. All image data of the specimens were loaded and analyzed by image analysis software, Virtual Slide System Ver.2.1 (Olympus Co., Tokyo, Japan).

 The CD-31-positive vessels were evaluated at the ulcer site, ulcer edge, and granulation tissue (×200) per specimen, and expressed as the number of blood vessels per field. VEGF-positive cells were evaluated with Virtual Slide System Ver.2.1 (×200; Olympus). *α*-SMA-positive areas were evaluated by image analysis software at the ulcer site, ulcer edge, and granulation tissue (×100) per specimen and expressed as the area per field.

### 2.6. Protein Preparation and Western Blotting

We examined the protein expression of inflammatory cytokines— monocyte chemotactic protein-1 (MCP-1), interleukin-1*β* (IL-1*β*), and tumor necrosis factor-*α* (TNF-*α*), angiogenesis growth factors; vascular endothelial growth factor (VEGF) and platelet derived growth factor (PDGF), and extracellular matrix remodeling factors; *α*-smooth muscle actin (*α*-SMA), transforming growth factor-*β* 1 (TGF-*β1*), basic fibroblast growth factor (bFGF), collagen III, and collagen I, in the area between the surface skin of the wound and the fascia of hamstring muscles by Western blotting. Skin tissues of the ulcer portion at days 3, 7, 14, and 35 were, respectively, homogenized in lysis buffer [137 mM NaCl, 20 mM Tris-HCl (pH 7.5), 1% polyoxyethylene octylphenyl ether (NP-40), 10% glycerol, 1 mM phenylmethylsulfonyl fluoride (PMSF), Protease Inhibitor Complete Mini (F-Hoffman-La Roche, Basel, Switzerland)] by using Kinemachika Polytron Homogenizer PT20SKR (Kinemachika AG, Lucerne, Switzerland). The lysates were prepared by centrifugation at 5,500 rpm for 10  min at 4°C, and the supernatants were collected. The concentration of protein in the supernatants was detected by Protein Assay Kit (Bio-Rad Laboratories, Hertfordshire, UK), reduced to 1 *μ*g/*μ*l with sodium dodecyl sulfate (SDS) sample buffer and distilled water, and then the sample was boiled for 4 min at 95°C. The samples were separated by 12% SDS-polyacrylamide gel electrophoresis and transferred to poly vinylidene difluoride (PVDF) membranes (Millipore Corp.). The membrane was treated with 5% skim milk with PBS-T for 1 h at room temperature, reacted with primary antibodies for Western blotting analysis overnight at 4°C, including MCP-1 (1 : 1000; Santa Cruz Biotechnology Inc.), IL-1*β*  (1 : 1000; Santa Cruz Biotechnology Inc.), TNF-*α* (1 : 1000; R&D Systems Inc., MN), VEGF (1 : 1000; Immuno-Biological Laboratories Co., Ltd., Gumma, Japan), PDGF (1 : 1000; Santa Cruz Biotechnology Inc.), *α*-SMA (1 : 1000; DAKO Cytomation Colorado Inc. Co., CO), TGF-*β1* (1 : 1000; Santa Cruz Biotechnology Inc.), bFGF (1 : 500; Wako Pure Chemical Industries, Osaka, Japan), collagen III (1 : 1000; Santa Cruz Biotechnology Inc.), and collagen I (1 : 1000; Santa Cruz Biotechnology Inc.). *β*-actin (Abcam, Cambridge, UK) was used as internal control. The antibodies were detected using horseradish peroxidase-conjugated anti-mouse, or anti-rabbit IgG (GE Healthcare UK, Buckingham, UK) for 1 h at room temperature and visualized by ECL Western blotting detection reagent (GE Healthcare) on X-ray film (RX-U. Fuji Safelight Glass No. 8U: Fujifilm, Tokyo, Japan) and determined quantity by Image J 1.34s software (National Institutes of Health, USA).

### 2.7. Statistical Analysis

All values were presented as mean ± SE. One-way analysis of variance (ANOVA) followed by Dunnett's test was used to determine the differences among groups on the assessments of ulcerated area, histological findings, and Western blotting. *P* < .05 was accepted as statistically significant. Statistical analyses were carried out using JMP 8.0 (SAS Institute, Cary, NC).

## 3. Results

### 3.1. Macroscopic and Histopathological Findings

**Figure 3:**
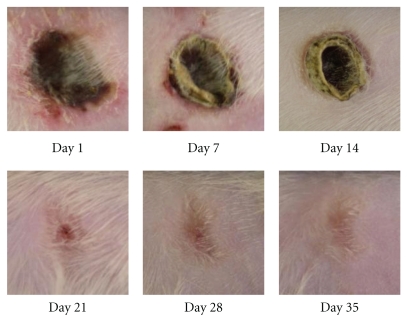
Typical macroscopic view of pressure ulcer of control group on days 1, 7, 14, 21, 28, and 35. Pressure-loaded portion became necrotic after release of loading (day 1), and crust was formed in the ulcer site with redness of surrounding skin (day 7). Size of ulcer gradually decreased (days 14, 21, and 28), and disappeared (day 35).

Immediately after completion of pressure loading, the skin surface was depressed and partially colored brown or white. After day 1 the pressure-loading portion became necrotic, and the skin surrounding the ulcer edge turned red and a crust was formed at the ulcer site. Gradually the skin ulcer decreased in size and then disappeared (Figure 3).

**Figure 4:**
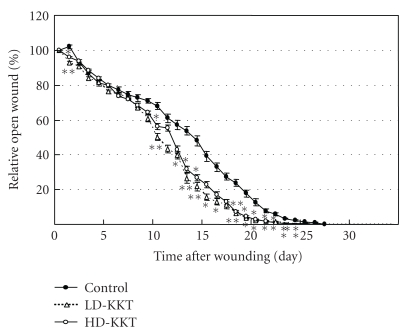
Time courses of the areas of pressure ulcers. Closed circles show the area of skin ulcer of control group, open triangles show that of the LD-KKT group, and open circles show that of the HD-KKT group. The areas of skin ulcers of the LD-KKT and HD-KKT groups were smaller than that of the control group on day 1, and there was no difference among the three groups on days 2 to 9. On day 10, days 12 to 16, and days 18 to 24, the areas of skin ulcers of the LD-KKT and HD-KKT groups were smaller than that of the control group. Values are expressed as mean ± SE (*n* = 6). *P < .05. **P < .01 versus control.

 The areas of pressure ulcers of the control, LD-KKT, and HD-KKT groups were evaluated each day. Those of the LD-KKT and HD-KKT groups on day 1 were significantly smaller than that of control, and there was no significant difference among them on days 2 to 9. On day 10, days 12 to 16, and days 18 to 24, the areas of the skin ulcers of the LD-KKT and HD-KKT groups were significantly smaller than that of the control group (Figure 4). The number of days until disappearance of skin ulcer in the control group was 26.67 ± 0.42 days, and those of the LD-KKT group (23.50 ± 3.35 days, *P* < .05) and HD-KKT group (22.33 ± 0.67 days, *P* < .01) were significantly shortened compared to the control group.

**Figure 5:**
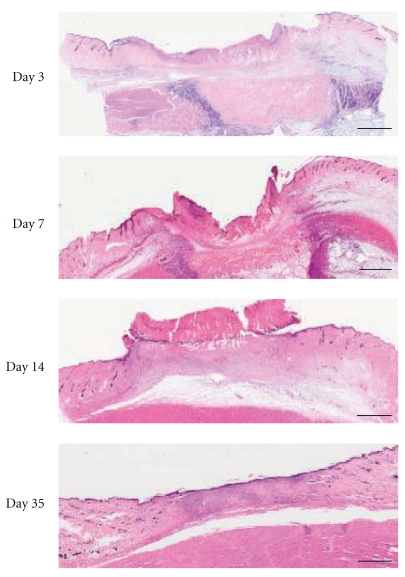
Histopathological findings of pressure ulcer. Typical microscopic views of HE stain of skin ulcer in the control group on days 3, 7, 14, and 35 are shown (scale bar, 2 mm).

 Histopathological findings of skin ulcer are shown in Figure 5. The base of skin ulcer was filled with a necrotic layer composed of fibrin and necrotic tissue. Although subcutaneous structures were viable, cutaneous muscles were broken and vessels lapsed into fibrinoid necrosis. Epidermal cells spread out from the ulcer edge to the ulcer site over time, and all ulcers disappeared by day 35. Epidermal cells of ulcer scar showed characteristics of a stratified squamous epithelium and then disappeared by day 35.

### 3.2. Immunohistochemical Findings

#### 3.2.1. CD-31

**Figure 6:**
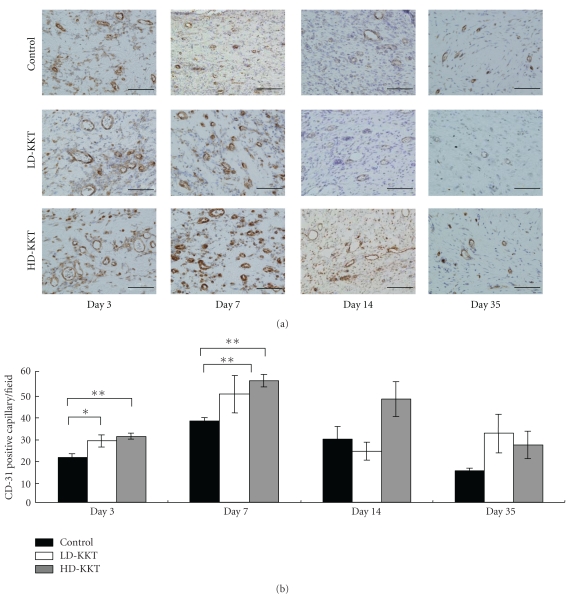
CD-31 immunohistochemical findings of vessels at pressure ulcer edge on days 3, 7, 14, and 35. CD-31 immunopositive vessels are stained brown ((a); scale bar, 100 *μ*m). The numbers of CD-31 immunopositive vessels per field (×200) were counted at wound tissue area including ulcer site, ulcer edge and granulation tissue (b). Values are expressed as mean ± SE (*n* = 4–7). *P < .05, **P < .01, versus control.

Positive reactions to anti-CD-31 antibody were observed at the ulcer edge and just below the part of pressure loading. At the ulcer edge, CD-31-positive vessels were observed in all groups after day 3, and more CD-31-positive vessels were seen in the LD-KKT and HD-KKT groups than in the control group. The numbers of CD-31-positive vessels of day 7 demonstrated an increasing tendency compared to those of day 3 in each group, and a lot of small vessels were seen on day 7. By day 14, the numbers of CD-31-positive vessels had decreased in all groups and then decreased further by day 35, becoming equal to normal skin not having undergone pressure loading (Figure 6(a)). The number of CD-31 immunopositive vessels per field (×200) was counted in the area of damaged tissue (ulcer site, ulcer edge, and granulation tissue), and on days 3 and 7 those of the LD-KKT and HD-KKT groups increased compared to that of the control group (Figure 6(b)).

#### 3.2.2. VEGF

**Figure 7:**
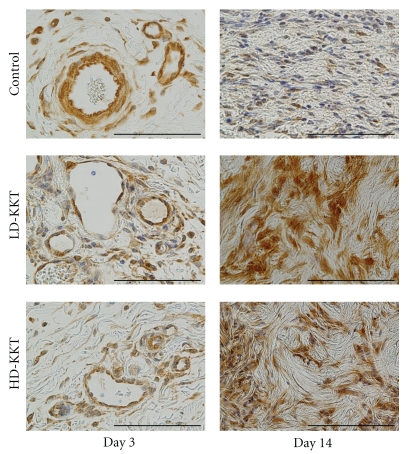
VEGF immunopositive area at pressure ulcer edge (days 3 and 14; scale bar, 100 *μ*m). On day 3, VEGF immunopositive cells were mainly endothelial cells. On day 14, the LD-KKT and HD-KKT groups had more VEGF immunopositive fibroblasts than the control group.

On day 3, positive reaction to anti-VEGF antibody was observed at the ulcer edge. The necrotic layer, granulation layer, and subcutaneous tissue of the central area showed negative reaction to anti-VEGF antibody, and there was no particular difference among the three groups. On day 14, numerous VEGF immunopositive cells were observed in the granulation tissue of all groups, and in KKT-administered groups more VEGF immunopositive cells were seen compared to the control group (Figure 7). On day 35, VEGF-positive cells were still observed in KKT-administered rats, but had almost disappeared in control rats.

#### 3.2.3. *α*-SMA

**Figure 8:**
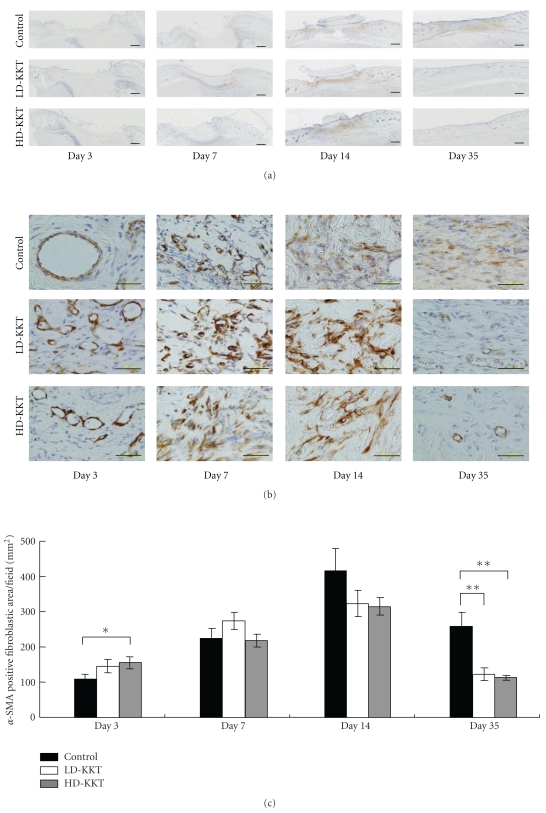
*α*-SMA immunohistochemical findings on days 3, 7, 14, and 35. *α*-SMA immunopositive cells in skin ulcer site are shown ((a); scale bar, 100 *μ*m). The numbers of *α*-SMA immunopositive cells per field were counted in the damaged area including ulcer site, ulcer edge and granulation tissue (b). All values are presented as mean ± SE (*n* = 4–7). *P < .05. **P < .01 versus control.

Positive reactions to anti-*α*-SMA antibody were observed at the ulcer edge and just below the portion of pressure loading. At the ulcer edge, vascular smooth muscle cells showed positive reaction to anti-*α*-SMA antibody in all groups from day 3. On day 7, positive reaction to anti-*α*-SMA was observed not only at vascular smooth muscle cells of subcutaneous tissue but also at myofibroblasts around granulated areas in connective tissue. On day 14, more *α*-SMA-positive myofibroblasts were seen in connective tissue, but on day 35 their number had decreased (Figure 8(a)). The images of stained whole fields of injured tissue, from the epidermis to the fascia of the hamstring, were scanned into the computer system, and the area of the positive reaction to anti-*α*-SMA antibody was measured by image analysis software. The average of the area of the positive reaction to anti-*α*-SMA antibody was calculated. The result showed that, on day 3, the area of the positive reaction to anti-*α*-SMA antibody in the HD-KKT group was significantly higher compared to control, on day 14 that of each group reached a peak, and on day 35 those in the LD-KKT and HD-KKT groups significantly decreased compared to that in the control group (Figure 8(b)).

### 3.3. Protein Levels by Western Blotting

**Figure 9:**
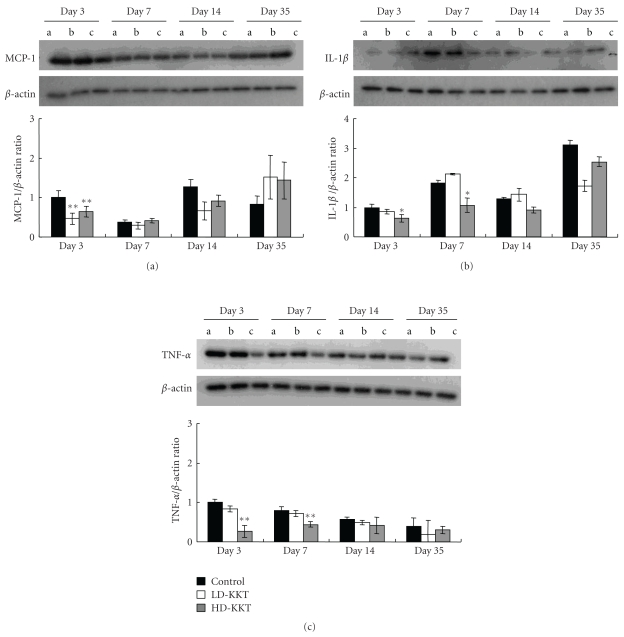
Expressions of inflammatory cytokines in skin tissue of pressure ulcer on days 3, 7, 14, and 35 as examined by Western Blotting. (a) MCP-1, (b) IL-1*β*, (c) TNF-*α*. Values are expressed as mean ± SE (*n* = 4–7). *P < .05, **P < .01 versus control.

Inflammatory cytokines: the protein levels of MCP-1 of the LD-KKT and HD-KKT groups were significantly lower than that the control group on day 3 (Figure 9(a)). The value of IL-1*β* in the HD-KKT group was significantly suppressed on days 3 and 7 compared to control (Figure 9(b)). TNF-*α* of the HD-KKT group was also significantly decreased compared to control on days 3 and 7 (Figure 9(c)).

**Figure 10:**
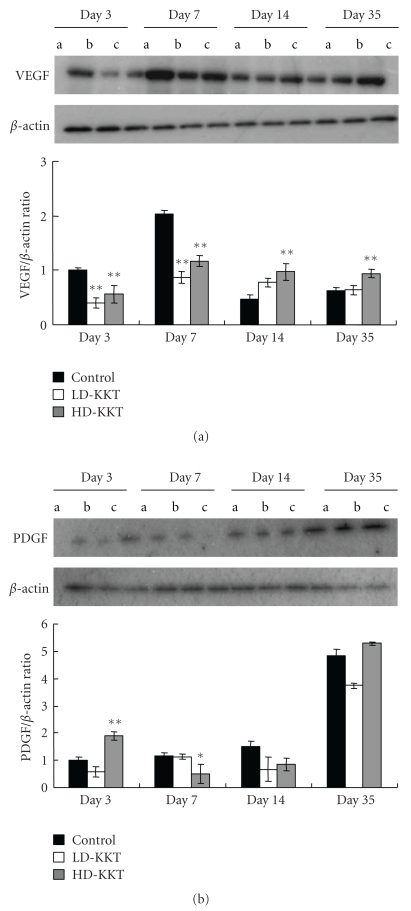
Expressions of angiogenesis growth factors in skin tissue of pressure ulcer on days 3, 7, 14, and 35 as examined by Western Blotting. (a) VEGF, (b) PDGF. Values are expressed as mean ± SE (*n* = 4–7). *P < .05, **P < .01 versus control.

 Angiogenesis growth factor: the protein levels of VEGF in the LD-KKT and HD-KKT groups were significantly lower than that of control on days 3 and 7, and that of the HD-KKT group was higher than that of control on days 14 and 35 (Figure 10(a)). PDGF of the HD-KKT group significantly increased compared to that of control on day 3 and decreased compared to that of control on day 7 (Figure 10(b)). 

**Figure 11:**
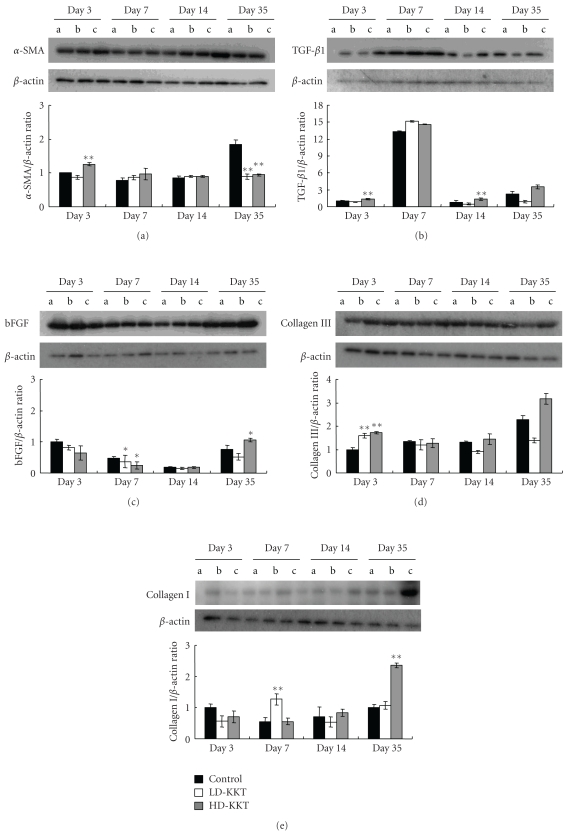
Expressions of extracellular matrix remodeling factors in skin tissue of pressure ulcer on days 3, 7, 14, and 35 as examined by Western Blotting. (a): *α*−SMA, (b): TGF-*β1*, (c): bFGF, (d): collagen III, (e): collagen I. Values are expressed as mean ± SE (*n* = 4–7). *P < .05, *P < .01 versus control.

 Extracellular matrix protein: the protein level of *α*-SMA of the HD-KKT group increased on day 3 and those of the LD-KKT and HD-KKT groups decreased on day 35 compared of that of the control group with statistical significance (Figure 11(a)). The values of TGF-*β*1 in the HD-KKT group on days 3 and 14 were significantly higher than that of control (Figure 11(b)). The values of bFGF in the LD-KKT and HD-KKT groups were significantly lower than that of control on day 7, and that of the HD-KKT group was higher than that of control on day 35 (Figure 11(c)). The protein levels of collagen III of the LD-KKT and HD-KKT groups were significantly higher than that of control on day 3 (Figure 11(d)). The protein level of collagen I of the LD-KKT group on day 7 and that of the HD-KKT group on day 35 were significantly elevated compared to those of control (Figure 11(e)).

## 4. Discussion

In the present study, we evaluated the effect of a Kampo formula, KKT, by a modified pressure ulcer rat model using a stainless steel cylinder. As a result, we clarified that KKT had a healing acceleration effect on pressure ulcer and some of its mechanism(s). By the previously reported method, rats under anesthesia received a heavy load for 24 h [15], but our modified method was able to develop pressure ulcer constantly with a relatively light load of pressure for 6 h daily for 4 days.

 In the present study, firstly from a macroscopic perspective, the number of days required for healing of pressure ulcer in KKT-administered groups was less than that of control. Further, we focused on the inflammatory phase, the early phase of wound healing, and analyzed inflammatory cell infiltration, but we could not find clear difference between KKT-administered rats and control rats in terms of pathological findings. We evaluated the protein expression of inflammatory cytokines such as MCP-1, IL-1*β*, and TNF-*α* by Western blotting, and we found that those in the KKT-administered groups were lower than that of control on days 3 and 7 in the early phase as a whole. It is known that the expression of inflammatory cytokines increases during the course of chronic wound [31, 32], so the possibility is suggested that KKT accelerates healing of pressure ulcer through the suppression of inflammatory cytokines.

 Next, we evaluated angiogenesis of the portion of pressure loading. By immunohistochemical examination, the number of vessels counted by using anti-CD-31 antibody, which labels vascular endothelial cells, was increased on days 3 and 7, so it is considered that KKT might increase angiogenesis in early phase. Further, anti-VEGF antibody-positive cells, which were mainly fibroblastic cells, were increased in KKT-administered rats compared to control especially on day 14. It is reported that VEGF promotes the increase of vascular permeability, angiogenesis, and storage of collagen, expresses highly under ischemic condition, and its peak is 3 to 7 days after wound injury [33]. In this study, the expression levels of VEGF were depressed in early phase (days 3 and 7) and elevated in late phase (days 14 and 35) in KKT-treated rats, and the level of PDGF, another angiogenesis factor, in the HD-KKT group increased on day 3 and decreased on day 7 compared to control. These variations of angiogenesis factors with the time course might be related to the acceleration of wound healing.

 The area of the positive reaction to anti-*α*-SMA antibody was assessed immunohistochemically to evaluate the accumulation of extracellular matrix. The area in the HD-KKT group in early phase was higher and that in late phase was lower compared to control. The protein expression of *α*-SMA showed similar findings to the histological study. The protein expression of TGF-*β*1, which is responsible for the phenotypic change from fibroblast to myofibroblast [34], was elevated on days 3 and 14. These results suggested the possibility that KKT induces TGF-*β*1 to transform from fibroblast to myofibroblast in the ulceration phase, and facilitates smooth restructuring of extracellular matrix in the tissue-remodeling phase.

 The protein expression of bFGF in the HD-KKT group was increased on day 35 in late phase. In this immunohistochemical study, the areas of the positive reaction to anti-*α*-SMA antibody in the KKT-administered groups were decreased on day 35. It is reported that bFGF inhibits the abnormal remodeling of extracellular matrix and is involved in the improvement of qualitative healing of wound by the induction of myofibroblast apoptosis [35–37]. Therefore, it is considered that KKT might have stimulated the transformation from fibroblast to myofibroblast in the early stage of the ulceration phase through increasing the protein level of TGF-*β*1 and might be associated with apoptosis of myofibroblast through the subsequent increase of the protein level of bFGF in late phase, then enhancing the remodeling of extracellular matrix. However, the mechanism of the increasing TGF-*β*1 and bFGF by KKT is not clear. These are the challenges to be addressed in future studies.

 As for extracellular protein, it is well known that collage III, reticular fiber, is produced with destruction of collagenous tissue and is replaced by collagen I, which gradually develops thick fiber [38]. In this study of KKT administration, the protein expression of collagen III was increased on day 3 in early phase, and that of collagen I was increased on day 35 in late phase. These results suggested the possibility that the increases of extracellular matrix proteins are associated with some of the mechanism(s) of the wound healing acceleration effect of KKT. In recent years, high-quality wound healing, in addition to fast healing, has been urgently needed from the treatment of pressure ulcer or skin ulcer [39]. It was reported that remodeling of extracellular matrix of acute wound required one to twelve months [40]. In the future, it will be necessary to demonstrate the histological findings after healing of skin ulcer by treatment with KKT. 

 Although the wound healing acceleration effects of each crude drug composing KKT and their representative compounds were not evaluated in this study, Paeoniae Radix showed wound healing acceleration effect in the *in vivo* experiment [41]. Additionally, it was also reported that the water extracts of Angelicae Radix and Astragali Radix showed angiogenic effects in the *in vitro* experiments through phosphorylation of p38 or JNK1/2 and VEGF-KDR/Flk pathway or P13K-Akt-eNOS pathway, respectively [42, 43]. It will be necessary to demonstrate the effects of each crude-drug component or active constituent in the future, as the effects of these crude-drug components might have been involved in the shortening effect of KKT on healing of pressure ulcer.

## 5. Conclusion

In the present study, the effects and the mechanism(s) of KKT, a traditional Japanese formula, were evaluated by using modified pressure-loading skin ulcer model of rats. The results revealed that KKT exerts a wound healing acceleration effect. Further, the possibility was raised that the effect of KKT might be associated with a decrease in certain cytokines, which are expressed in the inflammation phase of skin ulcer, increase of angiogenesis, and induction of extracellular matrix remodeling.

## References

[1] Sanada H, Nakagami G, Mizokami Y (2010). Evaluating the effect of the new incentive system for high-risk pressure ulcer patients on wound healing and cost-effectiveness: a cohort study. *International Journal of Nursing Studies*.

[2] Harrington C, Zagari MJ, Corea J, Klitenic J (2000). A cost analysis of diabetic lower-extremity ulcers. *Diabetes Care*.

[3] Kosiak M (1961). Etiology of decubitus ulcers. *Archives of Physical Medicine and Rehabilitation*.

[4] Knight SL, Taylor RP, Polliack AA, Bader DL (2001). Establishing predictive indicators for the status of loaded soft tissues. *Journal of Applied Physiology*.

[5] Bouten CV, Oomens CW, Baaijens FP, Bader DL (2003). The etiology of pressure ulcers: skin deep or muscle bound?. *Archives of Physical Medicine and Rehabilitation*.

[6] Macgregor L (2009). *International Guidelines. Pressure Ulcer Prevention: Prevalence and Incidence in Context. A Consensus Document*.

[7] Peirce SM, Skalak TC, Rodeheaver GT (2000). Ischemia-reperfusion injury in chronic pressure ulcer formation: a skin model in the rat. *Wound Repair and Regeneration*.

[8] Stadler I, Zhang RY, Oskoui P, Whittaker MS, Lanzafame RJ (2004). Development of a simple, noninvasive, clinically relevant model of pressure ulcers in the mouse. *Journal of Investigative Surgery*.

[9] Saito Y, Hasegawa M, Fujimoto M (2008). The loss of MCP-1 attenuates cutaneous ischemia-reperfusion injury in a mouse model of pressure ulcer. *Journal of Investigative Dermatology*.

[10] Peirce SM, Skalak TC, Rieger JM, Macdonald TL, Linden J (2001). Selective A_2A_ adenosine receptor activation reduces skin pressure ulcer formation and inflammation. *American Journal of Physiology*.

[11] Wassermann E, Van Griensven M, Gstaltner K, Oehlinger W, Schrei K, Redl H (2009). A chronic pressure ulcer model in the nude mouse. *Wound Repair and Regeneration*.

[12] Bosboom EMH, Bouten CVC, Oomens CWJ, Baaijens FPT, Nicolay K (2003). Quantifying pressure sore-related muscle damage using high-resolution MRI. *Journal of Applied Physiology*.

[13] Linder-Ganz E, Gefen A (2004). Mechanical compression-induced pressure sores in rat hindlimb: muscle stiffness, histology, and computational models. *Journal of Applied Physiology*.

[14] Stekelenburg A, Strijkers GJ, Parusel H, Bader DL, Nicolay K, Oomens CW (2007). Role of ischemia and deformation in the onset of compression-induced deep tissue injury: MRI-based studies in a rat model. *Journal of Applied Physiology*.

[15] Kaneko T, Hashimoto A, Umehara N, Tezukab M (2006). Effects of a new wound dressing material SG-01 in an experimental rat skin burn and decubitus ulcer model. *Journal of Health Science*.

[16] Clark RAF (1985). Cutaneous tissue repair: basic biologic considerations. I. *Journal of the American Academy of Dermatology*.

[17] Martinez-Ferrer M, Afshar-Sherif AR, Uwamariya C, De Crombrugghe B, Davidson JM, Bhowmick NA (2010). Dermal transforming growth factor-*β* responsiveness mediates wound contraction and epithelial closure. *American Journal of Pathology*.

[18] Detmar M, Brown LF, Schön MP (1998). Increased microvascular density and enhanced leukocyte rolling and adhesion in the skin of VEGF transgenic mice. *Journal of Investigative Dermatology*.

[19] Zhang JZ, Maruyama K, Iwatsuki K, Ono I, Kaneko F (1994). Effects of prostaglandin E_1_ on human keratinocytes and dermal fibroblasts: a possible mechanism for the healing of skin ulcers. *Experimental Dermatology*.

[20] Baumgartner I, Pieczek A, Manor O (1998). Constitutive expression of phVEGF165 after intramuscular gene transfer promotes collateral vessel development in patients with critical limb ischemia. *Circulation*.

[21] Thomas S, Banks V, Bale S (1997). A comparison of two dressings in the management of chronic wounds. *Journal of Wound Care*.

[22] Desneves KJ, Todorovic BE, Cassar A, Crowe TC (2005). Treatment with supplementary arginine, vitamin C and zinc in patients with pressure ulcers: a randomised controlled trial. *Clinical Nutrition*.

[23] Ellinger S, Stehle P (2009). Efficacy of vitamin supplementation in situations with wound healing disorders: results from clinical intervention studies. *Current Opinion in Clinical Nutrition and Metabolic Care*.

[24] Schols JMGA, Heyman H, Meijer EP (2009). Nutritional support in the treatment and prevention of pressure ulcers: an overview of studies with an arginine enriched oral nutritional supplement. *Journal of Tissue Viability*.

[25] Nagasaka K, Tosa H, Tasumi T, Shimada Y, Itou T (1998). Four cases report of intractable pressure ulcers effectively treated with Kigi-kenchu-to-ka-bushi. *Japanese Journal of Oriental Medicine*.

[26] Hikiami H, Sekiya N, Nagasaka K, Kouda K, Shimada Y, Terasawa K (2004). Case reports of diabetic foot successfully treated with Kigi-kenchu-to with additional ingredients. *Kampo Medicine*.

[27] Okamura N, Maki T, Ishida S (2000). Dissolution profiles of principal ingredients in Kampo medicinal powders by high-performance liquid chromatography. *Chemical and Pharmaceutical Bulletin*.

[28] Nogami M, Hoshi T, Kinoshita M, Arai T, Takama M, Takahashi I (2007). Vascular endothelial growth factor expression in rat skin incision wound. *Medical Molecular Morphology*.

[29] Chen BK, Leiferman KM, Pittelkow MR, Overgaard MT, Oxvig C, Conover CA (2003). Localization and regulation of pregnancy-associated plasma protein A expression in healing human skin. *Journal of Clinical Endocrinology and Metabolism*.

[30] Alizadeh N, Pepper MS, Modarressi A (2007). Persistent ischemia impairs myofibroblast development in wound granulation tissue: a new model of delayed wound healing. *Wound Repair and Regeneration*.

[31] Finnerty CC, Herndon DN, Przkora R (2006). Cytokine expression profile over time in severely burned pediatric patients. *Shock*.

[32] Mast BA, Schultz GS (1996). Interactions of cytokines, growth factors, and proteases in acute and chronic wounds. *Wound Repair and Regeneration*.

[33] Bao P, Kodra A, Tomic-Canic M, Golinko MS, Ehrlich HP, Brem H (2009). The role of vascular endothelial growth factor in wound healing. *Journal of Surgical Research*.

[34] Desmoulière A, Chaponnier C, Gabbiani G (2005). Tissue repair, contraction, and the myofibroblast. *Wound Repair and Regeneration*.

[35] Akasaka Y, Ono I, Yamashita T, Jimbow K, Ishii T (2004). Basic fibroblast growth factor promotes apoptosis and suppresses granulation tissue formation in acute incisional wounds. *The Journal of Pathology*.

[36] Akasaka Y, Ono I, Tominaga A (2007). Basic fibroblast growth factor in an artificial dermis promotes apoptosis and inhibits expression of *α*-smooth muscle actin, leading to reduction of wound contraction. *Wound Repair and Regeneration*.

[37] Akasaka Y, Ono I, Kamiya T (2010). The mechanisms underlying fibroblast apoptosis regulated by growth factors during wound healing. *The Journal of Pathology*.

[38] Mutsaers SE, Bishop JE, McGrouther G, Laurent GJ (1997). Mechanisms of tissue repair: from wound healing to fibrosis. *International Journal of Biochemistry and Cell Biology*.

[39] Shah M, Foreman DM, Ferguson MWJ (1992). Control of scarring in adult wounds by neutralising antibody to transforming growth factor *β*. *The Lancet*.

[40] Gurtner GC, Werner S, Barrandon Y, Longaker MT (2008). Wound repair and regeneration. *Nature*.

[41] Malviya N, Jain S (2009). Wound healing activity of aqueous extract of Radix paeoniae root. *Acta Poloniae Pharmaceutica*.

[42] Lam HW, Lin HC, Lao SC (2008). The angiogenic effects of Angelica sinensis extract on HUVEC in vitro and zebrafish in vivo. *Journal of Cellular Biochemistry*.

[43] Zhang Y, Hu G, Lin HC (2009). Radix Astragali extract promotes angiogenesis involving vascular endothelial growth factor receptor-related phosphatidylinositol 3-kinase/akt-dependent pathway in human endothelial cells. *Phytotherapy Research*.

